# Usefulness of indocyanine green fluorescence imaging to evaluate intestinal blood flow during laparoscopic surgery for strangulated small bowel obstruction: a report of two cases

**DOI:** 10.1016/j.ijscr.2025.111808

**Published:** 2025-08-14

**Authors:** Ryota Noguchi, Tsunehiko Maruyama, Takatomo Suzuki, Yusuke Ozawa, Reiji Nozaki, Tatsuya Oda

**Affiliations:** a Department of Surgery, Mito Saiseikai General Hospital, Ibaraki, Japan; b Department of Gastrointestinal and Hepato-Biliary-Pancreatic Surgery, Faculty of Medicine, University of Tsukuba, Ibaraki, Japan

**Keywords:** Strangulated small bowel obstruction, Indocyanine green, Laparoscopic surgery

## Abstract

**Introduction and importance:**

Strangulated small bowel obstruction (SBO) is an emergency condition in which intestinal necrosis can occur. However, it is sometimes difficult to precisely assess intestinal blood flow. In recent years, assessment of organ blood flow using indocyanine green fluorescence imaging (ICG-FI) has become increasingly common in laparoscopic surgery.

**Case presentation:**

We report two cases of strangulated SBO in which intestinal blood flow was assessed using indocyanine green and treatment was achieved in a minimally invasive manner through a laparoscopic approach. Case 1: A 76-year-old man presented with abdominal pain and was diagnosed with strangulated SBO. We performed emergency laparoscopic surgery and dissected the loop of the band into which the intestine was strangulated. Although the procedure was converted to open surgery, we used ICG-FI to evaluate the intestinal blood flow and decided to preserve the intestines. Case 2: An 86-year-old woman presented with vomiting and was diagnosed with strangulated SBO by using abdominal computed tomography. Emergency laparoscopic surgery was performed, and strangulation was relieved under a pneumoperitoneum. The intestine was preserved because intestinal blood flow was observed on the ICG-FI.

**Clinical discussion:**

In this report, we presented two cases of strangulated SBO in which laparoscopic surgery was initiated and ICG-FI was used intraoperatively. Traditionally, the decision to resect the bowel relies heavily on subjective findings, which can lead to unnecessary resections and postoperative complications. ICG-FI offers a more objective assessment of perfusion and has shown potential in various emergency and elective gastrointestinal procedures. Its intraoperative use can assist in real-time surgical decision-making, even in challenging cases with persistent discoloration after release of strangulation. Moreover, when laparoscopic access is feasible, this approach can enhance visualization, facilitate teamwork, and minimize invasiveness. While laparoscopy may not be appropriate in all cases, particularly in hemodynamically unstable patients or those with severe intestinal dilation, it remains a valuable strategy in selected patients, especially when combined with advanced tools such as ICG-FI.

**Conclusion:**

In laparoscopic surgery for strangulated small bowel obstruction, ICG fluorescence imaging can be a valuable tool for assessing intestinal blood flow, which can reduce unnecessary resection of the intestine and enhance patient outcomes in elective cases.

## Highlights

• ICG fluorescence imaging enabled real-time assessment of intestinal blood flow.
• ICG-FI helped avoid unnecessary bowel resection despite persistent discoloration.
• Minimally invasive approach improved recovery without major complications.
• ICG-FI provided objective perfusion assessment during emergency surgery.

## 1 Background

Strangulated small bowel obstruction (SBO) is an emergency in which intestinal necrosis can occur owing to impaired intestinal blood flow. Therefore, early diagnosis and appropriate surgical treatment are essential. Some patients may achieve recovery of intestinal blood flow after the release of strangulation. Traditionally, whether to conserve or resect the intestines has been based on factors such as vascular pulsation of the mesentery, the color tone of the intestinal wall, and peristalsis [1]. However, this is difficult to confirm in certain cases. A technique for evaluating organ perfusion using indocyanine green fluorescence imaging (ICG-FI) was established in the 1970s [2]. Laparoscopic surgery has been increasingly adopted to treat various gastrointestinal diseases, and it has recently been considered feasible for adhesive SBO in selected patients [[3], [4], [5]]. Additionally, there are some reports of emergency laparoscopic surgery for strangulated SBO, although the number of such reports is limited. In the present report, we describe two cases of strangulated SBO in which intestinal blood flow was assessed using indocyanine green fluorescence imaging, and treatment was achieved in a minimally invasive manner through a laparoscopic approach.

## 2 Case presentation

### 2.1 Case 1

**Fig. 1:**
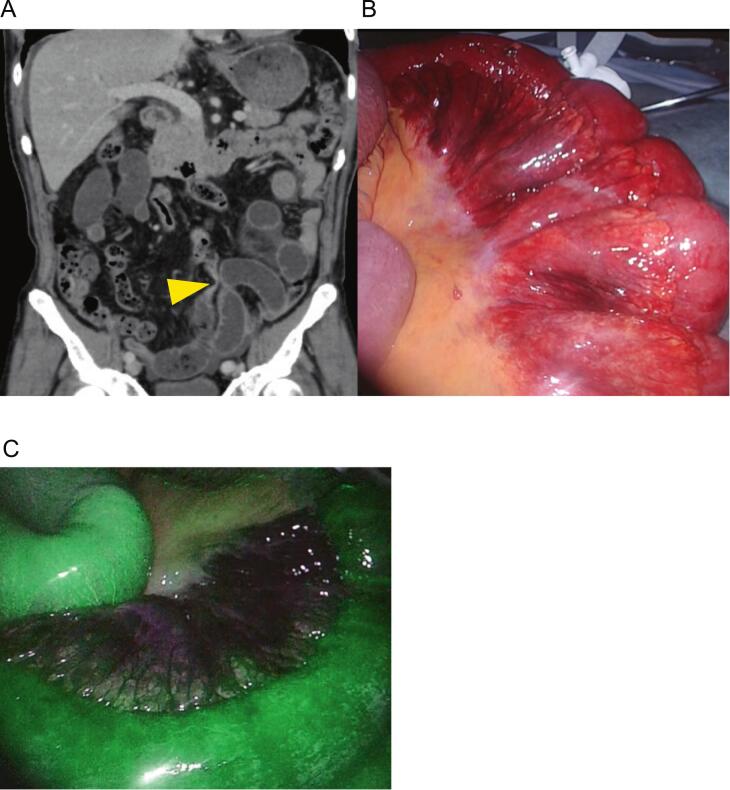
CT and intraoperative findings of case 1. **A** CE-CT findings on arrival (coronal view) of case 1. A closed loop sign is observed and the yellow arrowhead denotes the strangulated origin. **B** Intraoperative findings. A part of the small bowel displays a dark red color even after the strangulation was released. **C** Fluorescence was observed in the ileum. (For interpretation of the references to color in this figure legend, the reader is referred to the web version of this article.)

A 76-year-old man with a history of surgical treatment for appendicitis presented with abdominal pain. Physical examination revealed abdominal distension and mild tenderness throughout the abdomen. Blood tests revealed erythrocythemia (with RBC 5.85 × 106/μL), suggesting mild dehydration, and a minimal inflammatory reaction indicated by a white blood count of 19,000 cells/μL and C-reactive protein of 0.2 mg/dL. Abdominal contrast-enhanced computed tomography on the day of arrival revealed a closed-loop SBO, and strangulation was suspected (Fig. 1A). Emergency laparoscopic surgery was performed using three ports: a 12-mm umbilical port, a 5-mm left upper quadrant port, and a 5-mm left lower quadrant port. Laparoscopy revealed a fibrous band formation between the fatty appendices of the sigmoid colon and mesentery, with the small bowel strangulated into the loop of the band. The band was laparoscopically dissected, and strangulation of the small bowel was relieved. The small intestine is extensively dilated, making it difficult to secure a working space under the pneumoperitoneum. Therefore, we performed a mini-laparotomy and exteriorized a part of the small intestine to assess viability. However, judging the viability of the intestines based on their color was challenging (Fig. 1b). To assess the blood flow of the intestines, we intravenously injected indocyanine green at a dose of 0.25 mg/kg based on body weight; fluorescence was observed in the ileum 30 s after the injection (Fig. 1c). Therefore, we decided to preserve the intestines. The total operative time was 80 min, and postoperative paralytic ileus resolved after 7 days of conservative treatment. The patient was discharged on postoperative day 13.

### 2.2 Case 2

**Fig. 2:**
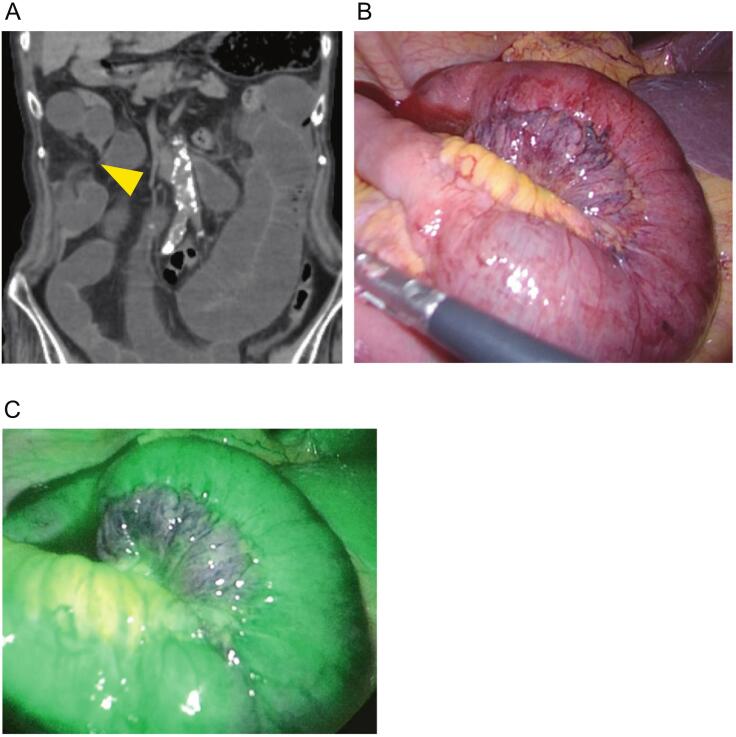
CT and intraoperative findings of case 2. **A** CE-CT findings on arrival (coronal view) of case 2. The yellow arrowhead denotes the strangulated origin. **B** Intraoperative findings. The color tone of the intestinal wall remained reddish even after the strangulation was released. **C** Fluorescence was observed in the ileum 20 s after the injection of ICG. (For interpretation of the references to color in this figure legend, the reader is referred to the web version of this article.)

An 86-year-old woman experienced vomiting and diarrhea for three days. The patient had no history of abdominal surgery or injury. No peritoneal irritation was observed. Blood tests indicated a mild inflammatory response with a white blood cell count of 9500 cells/μL and a C-reactive protein level of 5.49 mg/dL. Her blood urea nitrogen was 30.8 mg/dL, suggesting dehydration. Abdominal CE-CT showed ascites around the liver and in the Douglas pouch as well as dilation of the small intestine and a closed loop, indicating a strangulated SBO (Fig. 2a). Emergency laparoscopic surgery was performed using three ports: a 12-mm umbilical port, a 5-mm left upper quadrant port, and a 5-mm lower midline port. During laparoscopy, hemorrhagic ascites and adhesion of the omentum to the mesentery of the small intestine were observed, forming a loop where the small intestine was strangulated. The strangulation was relieved by transecting the omentum. However, the intestine remained slightly discolored even after the release of strangulation (Fig. 2b); therefore, we administered indocyanine green intravenously at a dose of 0.25 mg/kg and observed fluorescence in the intestine 20 s after injection (Fig. 2c). Consequently, the intestines were not resected. The entire procedure lasted 37 min. After surgery, the patient experienced a temporary reduction in swallowing function and resumed oral intake on postoperative day 8. The patient was transferred to the referral hospital on postoperative day 16.

## 3 Discussion

We report two cases of strangulated SBO in which the laparoscopic approach was initiated and intestinal blood flow was evaluated intraoperatively using ICG-FI. In both cases, it was difficult to determine whether to conserve or resect the intestine based on its color; observation of the intestine through a mini-laparotomy was necessary in one case, and ICG-FI confirmed that blood flow was ultimately adequate. As a result, the intestines were preserved in both cases, and postoperative recovery occurred without any significant adverse events such as small bowel stricture or perforation.

In this report, we present two cases in which, mild discoloration of the bowel remained after release of strangulation, the use of indocyanine green (ICG) fluorescence imaging enabled objective assessment of intestinal perfusion. This allowed for preservation of the bowel without resection, ultimately resulting in a favorable postoperative course. The application of ICG fluorescence imaging in cases of strangulated small bowel obstruction remains relatively uncommon in the literature. Furthermore, our report highlights the feasibility and benefits of combining laparoscopic release of strangulation with intraoperative ICG fluorescence evaluation in emergency settings. Through this case, we were able to clearly demonstrate the safety and minimally invasive nature of this approach.

In cases of strangulated SBO, the decision to resect or preserve the intestines after releasing the strangulation has traditionally been made based on factors such as vascular pulsation and the color tone of the intestines. However, even if there is no complete necrosis, intestinal discoloration often leads to the choice of intestinal resection. Standard clinical judgment alone leads to extensive bowel resection [6], with approximately 46 % of bowel resections being unnecessary [7]. Bowel resection significantly affects postoperative complications and quality of life [8]. The use of ICG-FI, which utilizes a near-infrared camera, has shown promise in evaluating intestinal blood flow [2]. This method has been used in various digestive surgeries, including laparoscopic colorectal cancer surgery [9], and is useful for emergency surgeries such as hernia incarceration [10] and non-occlusive mesenteric ischemia [11]. We used ICG-FI to evaluate intestinal blood flow in cases of strangulated SBO, where intestinal discoloration persisted even after the release of the strangulation. As a result, we were able to preserve the intestines, and the patients showed significant progress in recovery.

One benefit of ICG-FI is that it provides an objective indicator of intestinal blood flow, unlike subjective assessments such as vascular pulsation of the mesentery and color tone of the intestinal wall. Additionally, ICG-FI can be monitored by multiple surgeons during surgery. In cases where laparoscopic surgery is conducted and intestinal resection is not necessary, blood perfusion evaluation using ICG allows for smaller incisions and less invasive procedures because it does not rely on tactile sensation.

Although ICG-FI is applicable in both open and laparoscopic surgeries, its role is especially crucial in laparoscopic procedures where tactile assessment, such as detecting mesenteric pulsations, is not possible. In open surgery, ICG-FI also provides valuable objective data to supplement traditional subjective evaluations. However, in the laparoscopic setting, ICG-FI can compensate for the absence of direct palpation, offering real-time visualization of perfusion and enabling safer decision-making regarding bowel preservation.

Laparoscopic surgery is a minimally invasive and effective treatment method for strangulated SBOs. Recently, the number of facilities performing this procedure has increased. One report stated that in 53 cases of strangulated SBO in which intestinal resection was ultimately not performed, the group that initially opted for laparoscopic surgery experienced better outcomes in terms of time to oral intake after surgery and postoperative analgesic use than the group that initially opted for open surgery [12]. The reasons for the shorter time to oral intake are thought to include less stimulation to the intestines, smaller incisions promoting early mobilization, and contributing to the early return of bowel peristalsis. Regarding the time from surgery to ingestion, the superiority of laparoscopic surgery is thought to originate from less irritation to the intestine, resulting in early recovery of bowel movements. In addition, a smaller incision promotes early ambulation, which contributes to early recovery from peristalsis.

Laparoscopic surgery for strangulated SBO is an effective option; however, it is difficult to determine whether it applies to all cases. One report stated that laparoscopic surgery is not suitable for patients with preoperative hemodynamic instability [13]. It is also said that in cases with dilated intestines, the risk of bowel injury is high, and especially when it is difficult to secure sufficient working space, immediate conversion to open surgery should be considered [14].

However, we acknowledge that laparoscopic surgery may not be suitable in all cases, particularly when preoperative imaging reveals marked small bowel dilation. At our institution, we carefully assess preoperative CT scans to evaluate the extent of intestinal distension and to estimate the location of strangulation. If it is judged that a safe working space cannot be secured laparoscopically, or that there is a high probability of requiring conversion, we may elect to proceed directly with open surgery from the outset. Surgical approach decisions are made with patient safety as the highest priority.

One report stated that, in 43 cases of strangulated SBO in which bowel resection was performed, there was no statistical difference in the postoperative length of stay, time from surgery to ingestion, or incidence of postoperative complications between the initial laparoscopic surgery and open surgery groups. If an extended area of the strangulated intestine develops irreversible ischemic changes, handling it under laparoscopy and pulling it through a mini-laparotomy becomes challenging. However, when irreversible ischemic change is limited to a small area, performing a mini-laparotomy, bowel resection, and extracorporeal anastomosis is a feasible approach [15].

In addition, it is important to emphasize that ICG-FI confirms macrovascular perfusion but does not guarantee tissue viability. In cases of prolonged strangulation, the mucosa and submucosa may be irreversibly damaged even if the serosal surface appears well-perfused. Previous experimental and clinical observations have reported delayed complications such as ulceration or perforation in segments with good fluorescence. Therefore, ICG-FI should be used as a tool to confirm perfusion, not to definitively assess viability.

A multimodal approach is essential for intraoperative evaluation, including:

1) Visual inspection (color, peristalsis, wall thickness),
2) Palpation (turgor),
3) ICG fluorescence (perfusion),
4) Observation over time (e.g., reassessment after 20–30 min), and.
5) Second-look surgery when uncertainty remains.

Such comprehensive assessment provides a more reliable basis for surgical decision- making and may prevent both unnecessary resections and delayed complications.

It is important to evaluate the presence of irreversible bowel ischemia not only based on physical examination but also by referring to preoperative CT scans to determine the suitability of laparoscopic surgery [16]. However, it is not clear whether extensive bowel ischemia can be accurately diagnosed preoperatively. Diagnostic laparoscopy is an option to determine the applicability of laparoscopic surgery [17]. A laparoscopic observation strategy to assess the presence of necrotic bowel with ICG-FI and the extent of intra-abdominal space, and then decide whether to proceed with open surgery, may also be an effective approach.

At our facility, we have adopted the approach of initially starting laparoscopic surgery in cases of strangulated ileus in which the patient's vital signs were stable. Although laparoscopic surgery for strangulated SBO is not applicable in all cases, it has the potential to achieve favorable postoperative outcomes. As demonstrated in the current cases, the combined use of ICG to evaluate intestinal blood flow enabled a safe decision to preserve the intestine and allowed for minimally invasive treatment.

## 4 Conclusion

In laparoscopic surgery for strangulated SBO, ICG-FI can be a valuable tool for assessing intestinal blood flow, which can reduce unnecessary resection of the intestine and enhance patient outcomes in elective cases.

## 5 SCARE guidelines

This work has been reported in accordance with the SCARE criteria (Surgical Case Report Guidelines) [18].

## Consent for publication

Informed consent was obtained from the patients for this case report.

## Ethical approval

The study was reviewed and deemed exempt from full ethical review by the Ethics Committee.

## Funding

No specific grant was received from any funding agency in the public, commercial, or non-profit sectors.

## Author contribution

All authors contributed to the study's conception and design of this study. The material preparation, data collection, and analyses were performed by Noguchi and Maruyama. The first draft of the manuscript was written by Ryota Noguchi, and all authors commented on previous versions of the manuscript. All the authors have read and approved the final version of the manuscript.

## Guarantor

Dr. Tsunehiko Maruyama is the guarantor of this case report and accepts full responsibility for the work and the decision to publish.

## Research registration number

This case report is not a ‘First in Man’ study and therefore does not require registration.

## Conflict of interest statement

There is no conflict of interest regarding the publication of this case report.

## Data Availability

The datasets used and/or analyzed during the current study are available from the corresponding author on reasonable request.
